# Physical Activity as an Imperative Support in Breast Cancer Management

**DOI:** 10.3390/cancers13010055

**Published:** 2020-12-28

**Authors:** Miguel A. Ortega, Oscar Fraile-Martínez, Cielo García-Montero, Leonel Pekarek, Luis G. Guijarro, Alejandro J. Castellanos, Lara Sanchez-Trujillo, Natalio García-Honduvilla, Melchor Álvarez-Mon, Julia Buján, Álvaro Zapico, Guillermo Lahera, Miguel A. Álvarez-Mon

**Affiliations:** 1 Unit of Histology and Pathology, Department of Medicine and Medical Specialities, Faculty of Medicine and Health Sciences, University of Alcalá, 28801 Alcalá de Henares, Spain; oscar.fraile@edu.uah.es (O.F.-M.); cielogar@ucm.es (C.G.-M.); leonel.pekarek@edu.uah.es (L.P.); alejandro.jimenezc@edu.uah.es (A.J.C.); lstrujillo@salud.madrid.org (L.S.-T.); natalio.garcia@uah.es (N.G.-H.); melchor.alvarezdemon@uah.es (M.Á.-M.); mjulia.bujan@uah.es (J.B.); guillermo.lahera@uah.es (G.L.); miguelangel.alvarezm@edu.uah.es (M.A.Á.-M.); 2 Ramón y Cajal Institute of Sanitary Research (IRYCIS), 28034 Madrid, Spain; 3 Cancer Registry and Pathology Department, Hospital Universitario Principe de Asturias, 28806 Alcalá de Henares, Spain; 4 University Center for the Defense of Madrid (CUD-ACD), 28047 Madrid, Spain; 5 Unit of Biochemistry and Molecular Biology (CIBEREHD), Department of System Biology, University of Alcalá, 28801 Alcalá de Henares, Spain; luis.gonzalez@uah.es; 6 Immune System Diseases-Rheumatology, Oncology Service an Internal Medicine, University Hospital Príncipe de Asturias, (CIBEREHD), 28806 Alcalá de Henares, Spain; 7 Department of Surgery, Medical and Social Sciences, Faculty of Medicine and Health Sciences, University of Alcalá, 28801 Alcala de Henares, Spain; alvaro.zapico@salud.madrid.org; 8 Obstetrics and Gynecology Service, Center for Biomedical Research in the Mental Health Network, University Hospital Príncipe de Asturias, 28806 Alcalá de Henares, Spain; 9 Psychiatry Service, Center for Biomedical Research in the Mental Health Network, University Hospital Príncipe de Asturias, 28806 Alcalá de Henares, Spain; 10 Department of Psychiatry and Medical Psychology, Hospital Universitario Infanta Leonor, 28031 Madrid, Spain

**Keywords:** breast cancer, physical activity, supportive therapy, lifestyle intervention, breast cancer prevention and management

## Abstract

**Simple Summary:**

During the recent years, the field of exercise and physical activity have expanded prominently, not only for the prevention but also for the management of different pathologies, including breast cancer. A broad range of studies have been conducted to analyze this relationship, in aim to find the best routines and use of exercise in breast cancer management. Although many works provide contradictory results in these terms, there is less doubt regarding the role of physical activity as a central support for breast cancer patients, due to the systemic benefits of exercise in these women, therefore decreasing the risk of breast cancer-related complications and increasing their quality of life and survival. Future research should be established in order to individualize the physical activity performed by these women based on the available scientific evidence in a multidisciplinary context to maximize the success of breast cancer management.

**Abstract:**

Breast cancer (BC) is the most common malignancy and the second cause of cancer-related death among women. It is estimated that 9 in 10 cases of BC are due to non-genetic factors, and approximately 25% to 30% of total breast cancer cases should be preventable only by lifestyle interventions. In this context, physical activity represents an excellent and accessible approach not only for the prevention, but also for being a potential support in the management of breast cancer. The present review will collect the current knowledge of physical activity in the background of breast cancer, exploring its systemic and molecular effects, considering important variables in the training of these women and the evidence regarding the benefits of exercise on breast cancer survival and prognosis. We will also summarize the various effects of physical activity as a co-adjuvant therapy in women receiving different treatments to deal with its adverse effects. Finally, we will reveal the impact of physical activity in the enhancement of quality of life of these patients, to conclude the central role that exercise must occupy in breast cancer management, in an adequate context of a healthy lifestyle.

## 1. Introduction

Breast cancer (BC) is the most common malignancy affecting females and the second cause of cancer-related death in this population, where 1 in 8 women are at risk to develop BC [1,2]. Although frequently diagnosed in postmenopausal women of 60–65 years old [3,4], BC can also affect premenopausal young women under 40 [5] and even men [6]. Approximately 5% to 10% of BC cases are considered hereditary, importantly affecting these two last groups, mainly due to BRCA1/2 mutations [7,8]. The remaining cases are attributable to reproductive factors, directly related to hormonal levels such as early menarche, later menopause, nulliparity, or the use of oral contraceptive pills and modifiable risk factors like obesity, alcohol consumption, sedentarism, diet, or smoking [9,10].

BC may be classified in different ways. Depending on its site, it could be considered as non-invasive/in situ tumors or invasive/infiltrating tumors. TNM stratification (Tumor Nodes and Metastases) analyzing tumor size, lymph nodes affected, and metastasis (Stages 0–IV) may also have important consequences in establishing a prognosis of the disease [11,12]. Notwithstanding, molecular stratification of breast tumors has provided the most important advances in the knowledge of BC biology and prognosis. Thus, BC cells may be divided according to the presence/absence of estrogen and progesterone receptor (ER/PR), human epidermal growth factor receptor 2 (HER2 or ErbB2) and Ki67 status are used to identify the tumors into luminal A (ER/PR-positive/HER2-negative/low Ki-67), luminal B (ER- and/or PR-positive/HER2 absent/high Ki-67), HER2-positive luminal B (ER- and/or PR-positive/HER2 overexpression/any Ki-67), non-luminal HER2-positive (ER and PR negative/HER2 overexpression), and triple-negative breast cancer (or TNBC) (ER and PR and HER2-negative), the last group being those with the worst prognosis [13].

Different strategies are conducted when a patient develops BC, depending on the diagnosis. For instance, lobular carcinoma in situ does not need treatment, whereas ductal carcinoma in situ requires breast-conserving surgery and radiation therapy [14]. Moreover, choice of systemic therapies such as adjuvant and neoadjuvant therapies frequently represent important procedures, depending on the lymph involvement, menopausal status, or BC subtype [15]. Among them, endocrine therapy like aromatase inhibitors (AI) or Herceptin (Trastuzumab) are prominent approaches for luminal and HER2-enriched subtypes respectively [16], whereas for aggressive TNBC tumors, immunotherapy together with novel molecular targets are being used, including microRNAs, PARP (Poly ADP-ribose polymerase), or PI3K (Phosphoinositide 3-kinases) inhibitors [17,18,19]. Thus, advances in the therapies provided for the patients, along with early diagnosis and screening, have provoked a noteworthy reduction in overall cancer mortality and cancer-specific survival during the last decades in Europe [20].

On the other hand, there is still a long road to cover. Being diagnosed with BC as well as the treatment received has a strong impact on the lives of these women, including in their self-image, sexual health, psychological attitudes, and psychiatric disorders such as anxiety or depression, even in the BC survivors [21,22,23]. For that reason, a multidisciplinary management is needed in patients with BC, not only from medical care but also considering the quality of life (QOL) of these women, which instead will positively influence BC-related survival and a successful management [24].

In this great context, physical activity (PA) has been shown to present a broad spectrum of benefits in cancer management, reducing fatigue, muscular weakness, or functional capacity, among other effects, improving the QOL of BC patients, contributing to physical and mental well-being during the therapy received [25,26]. The present review will collect the current knowledge of exercise in the background of BC, with the aim to explore its multiple links with this condition along with current and potential uses of PA in these women.

## 2. PA in the Prevention of BC

PA could be defined as any corporal movement conducted by skeletal muscles which involves energy expenditure [27]. PA represents a pivotal tool in prevention of BC, partly due to its associated increased global DNA methylation [28]. The consequences of PA in the body of BC patients will be subsequently unraveled. Firstly, there is consistent evidence that up to 90–95% of total cancer cases are related to lifestyle and environmental factors, opening potential windows of opportunities to preventive measures, at least following the minimum established recommendations for diet, exercise, and weight control [29]. In fact, experts report that approximately 1 in 4 of total BC cases could be preventable only by successful lifestyle management [30], and previous studies showed us that there is a 25% average risk reduction to develop BC in active women when compared with non-active women [31].

**Figure 1:**
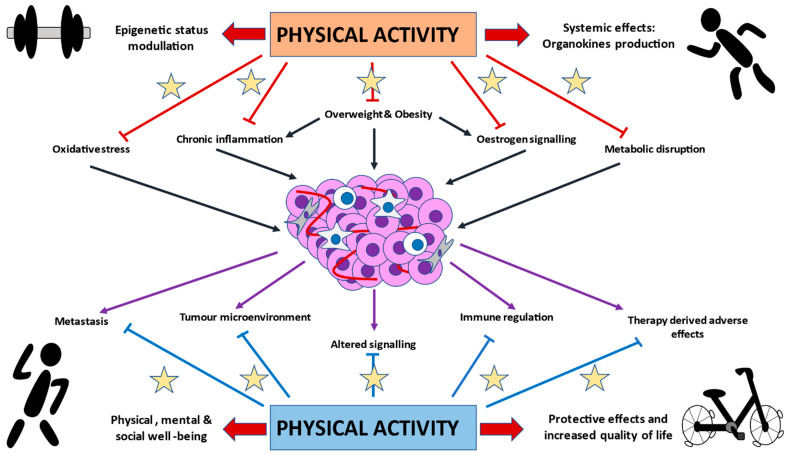
Graphical representation of the potential effects of physical activity (PA) in breast cancer (BC). As it is appreciated, PA may play a prominent role in the prevention of BC through its effects in important carcinogenic drivers, including oxidative stress, chronic inflammation, overweight/obesity, estrogen, and dysmetabolism. The main mechanisms are derived from epigenetic modulatory properties of PA and its systemic effects, including the production of organokines (such as myokines, adipokines, batokines, hepatokines, etc.). On the other hand, once BC is established, PA may also confer substantial benefits for BC patients, through its inhibitory effects on metastasis, molecular signaling, immune dysregulation, or tumor-favoring microenvironment. What is more, PA represents a potential tool to reduce therapy-derived adverse effects, due to its protective capacities and role in the physical, mental, and social well-being, leading to an increased quality of life of BC patients.

Premenopausal women appear to show higher benefits from PA than postmenopausal women, in which superior doses of PA are needed to obtain the same results [32,33]. However, it changes in patients with familiar history of BC, where PA appears to be less effective in premenopausal but important in postmenopausal women [34]. Age is also an important factor to consider in the risk reduction of BC associated with PA. Thus, the protective effects of PA at different ages are about 16% during adolescence, 8% for early adulthood, 15% for middle adulthood, and 17% for women aged 50 and above [35]. This could have important implications, as an increased trend of young women under 40 presenting BC has been reported [36] and PA may provide less benefits in the youngest adult women, therefore demonstrating the need of finding and combining PA with additional effective preventive measures in this group of people. Moreover, early life seems to be a fundamental window of susceptibility to reduce breast cancer risk, especially from 5 to 19 years old [37]. In this line, Lammert et al. [38] described a risk reduction of up to 38% of BC in active women who were BRCA1/2 carriers between 12 to 17 years old, compared with non-active women. Conversely, another study only found this association in women with a high average time between menarche and first pregnancy (20 years) [39], thereby showing the contradictory data regarding PA in the prevention of BC. However, because of the multiple benefits from practicing PA, it is undeniable that it should be recommended from infancy and sustained during the whole life of women (Figure 1).

A meta-analysis conducted by Wu et al. [40] demonstrated that the risk of BC decreased by 2% for every 25 metabolic equivalent (MET) MET-h/week of non-occupational activities (equivalent to 10 h/week), by 3% for every 10 MET-h/week (equivalent to approximately 2 h/week walking at 3.2 km/h or 1 h/week running at 9–10 km/h), and by 5% for every 2 h/week increment in moderate plus vigorous recreational activity. In this line, Kyu et al. [41] described that low active (600–3999 MET minutes), moderately active (4000–7999 MET minutes), and highly active women (≥8000 MET minutes) reduced risk of BC by 3%, 6%, and 14%, respectively. Chan et al. [42] also observed that vigorous activity was more strongly associated with reduced relative risk (RR) in premenopausal (RR = 0.79) than postmenopausal (RR = 0.90) women, although postmenopausal women were more sensitive to total, recreational, and occupational PA compared to premenopausal women. These studies suggest the major influence of PA in the prevention of BC, and minimum recommendations should be followed by everybody, although it seems that the higher the dose or intensity of exercise, the more protection against BC.

## 3. Exploring the Biological and Molecular Basis of PA in BC Development and Progression

The highly notorious impact of PA in BC is mainly due to their convergence and opposite effects into many systemic factors, including oxidative stress, immune regulation, or through sexual and metabolic hormones, hence reducing the risk of BC [43]. Likewise, PA may interact directly with the tumor and its environment, therefore being inversely correlated with the establishment and progression of all the subtypes of BC, including TNBC [44]. Here, we will summarize the different mechanisms of PA against the promotion and progression of BC.

### 3.1. PA and Sexual Hormones

One of the potential effects of PA in BC is through its action in the sexual hormones, whose levels are different depending on menopause status. Endogenous estrogens signaling have been shown to play a central role in the initiation and development of BC, particularly in postmenopausal women [45]. PA is a negative modulator of estrogen levels which aid to explain the preventive role of PA in these women [46,47]. Conversely, in the case of postmenopausal women using Hormone Replacement Therapy, favorable effects of PA in this group seem to be overridden [48]. Once BC is established, the action of estrogen signaling acquires further complexity as both oncogenic and antitumoral effects of estrogen have been reported, regulating some hallmarks of cancer like apoptosis/survival, proliferation, angiogenesis, and metastasis [49]. Whereas ovaries are the most important source of estrogen in premenopausal women, adipose tissue is the main producer in postmenopausal women [50], having a profound impact in ER-positive BC incidence [51]. It has been described that overweight and obesity directly influence estrogen signaling, prominently through promoting the expression and activity of the enzyme aromatase, ER expression and activation, along with estrogen production and bioavailability [52]. PA also has a head role in the adipose tissue, modulating its metabolism and fat mobilization [53]. In the case of premenopausal women, central obesity is closely associated with higher risk of TNBC, but not with luminal BC [54]. Although overweight and obesity are considered potential risk factors of BC in both premenopausal and postmenopausal women, the first group are better adapted to increased hormone levels. However, overweight and obesity are related with low-grade chronic inflammation, insulin resistance, hyperactivation of cell signaling pathways such as insulin-like growth factor (IGF), and adipose tissue dysregulation, which are, in fact, triggers of breast carcinogenesis [55]. What is more, visceral and intramuscular adiposity are also considered potential risk factors of cardiovascular complications after BC diagnosis [56]. Thereby, beneficial effects of PA in weight loss, body composition, and immune and metabolic parameters seem to be the most important action of exercise in BC prevention and management. 

### 3.2. Immunomodulatory Effects of PA

Exercise has been shown to be a key modulator of the immune system. On the one hand, it is known that PA displays powerful immunomodulatory and anti-inflammatory effects in the organism, regulating both immune populations and cytokines production [57,58]. As described by Hanahan and Weinberg [59], immune evasion and tumor-promoting inflammation are considered hallmarks or major characteristics of all cancer cells. In the case of BC, the inflammatory environment favored by the tumor promotes the activation of myeloid-derived suppressor cells (MDSCs), or T regs lymphocytes negatively modulate the activity of Cytotoxic T (CTL) and Natural Killer (NK) lymphocytes, two powerful antitumoral cells [60]. The main cytokines altered included Interleukins (IL) IL-1β and IL-6, and tumor necrosis factor-α (TNF-α) [61]. Multiple favorable changes in the immune system of BC patients have been reported due to PA, including in T helper lymphocytes and CTL populations, T regulatory cells, NKs, monocytes, tumor-associated macrophages, IL-6, and tumor necrosis factor (TNF-α) [62,63]. In addition, PA was associated with the reduction of systemic immunological markers, which may provide important implications in cancer cachexia [64]. Thus, PA may have opposite, beneficial effects in the immune system of women with BC. However, further clinical trials may be established to unravel the status and function of immune cells in patients with BC practicing PA [65].

### 3.3. Metabolic Effects of PA

PA interacts with a wide variety of metabolic hormones. This influence could be direct (Cortisol, aldosterone, estrogen, testosterone, D vitamin, etc.) or through secondary messengers (Insulin, endorphins, leptin, IGF, among others) [66]. Every person may respond differently to PA depending on the type, intensity, and duration of the exercise. Consequently, PA may regulate various key processes such as appetite, energy homeostasis, or insulin sensitivity, which may have important implications in BC [67,68,69]. However, high volume, excessive intensity, or overtraining could also lead to abnormal hormonal responses, which can be detrimental for the individual [66]. Also, PA stimulates the production of muscle cytokines, known as “myokines”, with important autocrine functions regulating muscle metabolism, together with paracrine and endocrine effects on a wide variety of organs and tissues, including bone, liver, or immune system [70]. Prominently, brain is another structure highly sensitive to PA and myokines are critical to the regulation of feeding centers, metabolism of kyneurine, or through the production of brain-derived neurotrophic factor (BDNF), with a pivotal role in neurogenesis, memory, and learning [71]. This could bring huge consequences for individuals’ mental health during and after treatment, as it will be later described. Apart from myokines, PA stimulates the production of hepatokines, osteokines, and adipokines, which may provide important effects in energy expenditure, fat mass loss, reduced inflammation, and weight control induced by exercise [72]. Batokines (brown adipose tissue cytokines) are also activated by cold or PA, which may have an interesting role in controlling the “browning” of white adipose tissue, reducing fat accumulation [73]. Likewise, PA has been shown to modulate gut microbiota, with important consequences in both systemic and metabolic effects [74]. Gut microbiota dysbiosis has also been observed in BC patients [75], although it is difficult to establish if it is a cause or consequence to the pathological environment. Metabolic effects of PA may be important not only in the prevention of BC but also providing a central support in BC management, directly interacting with other risk or prognostic factors.

### 3.4. PA and Oxidative Stress

Oxidative stress is another point of study in BC carcinogenesis. Oxidative stress occurs due to an imbalance between unstable oxidant molecules, reactive oxygen species (ROS) or reactive nitrogen species (RNS), and antioxidants (AOX), responsible for the scavenger of both ROS and RNS [76,77]. The increase of ROS promotes damage in lipids, proteins, and nucleic acids, where it produces DNA mutations and regulation of downstream products, which may contribute to tumorigenesis [78]. It is known that OS promotes the development and progression of BC tumoral cells, through the stimulation of malignant phenotype including death evasion, hyperproliferation, angiogenesis, invasiveness, and metastasis [79]. Importantly, estrogens may influence the ROS production, acting synergically through different signaling, promoting their carcinogenic effects [80]. PA may be helpful for the modulation of oxidative stress. Firstly, PA is associated with an increased DNA damage repair and AOX activity, also affecting the systemic oxidative status [81]. This could have important implications not only in the prevention of BC but also in the long-term QOL of BC survivors [82]. However, similar studies did not report any changes in systemic oxidative stress markers in postmenopausal women after one year of PA (225 min/week) on neither damage nor AOX markers [83]. This could be explained because, when combined with a proper diet, better results are obtained, thus elucidating the important role of an adequate diet and PA on the modulation of oxidative stress levels, which may prevent BC development [84]. On the other hand, tumoral cells express higher levels of ROS, but also of AOX molecules, hence demonstrating that BC cells are able to tightly control their oxidative status [85]. In fact, it is known that cancer cells are more susceptible to exogenous ROS variations, which may provide an important strategy to selectively target these cells [79]. Similarly, downregulation of ROS may also promote apoptosis and antitumoral effects [86]. Because of that, targeting this oxidative regulation provides a promising tool to kill tumoral cells. PA is associated with both antioxidant production and as a powerful inductor of oxidative stress and lipid peroxidation in BC, being able to disrupt this balance and behave as an important co-adjuvant during therapy [87]. Interestingly, genetic polymorphisms seem to play an important role in the pro-oxidant/antioxidant effect of PA [88], thus explaining different responses that women may have to PA leading to a greater or lower reduction of BC risk.

### 3.5. PA Direct Actions in BC Tumors

PA could also interact directly with tumor biology at different levels. On the one hand, it may play a vital role in metastasis prevention. PA leads to mechanical stimulation on the osteocytes, which may provide substantial improvement in bone health [89] along with the prevention of bone metastasis of BC cells [90]. To deepen the relation between PA bone and BC metastasis, a clinical trial is being conducted to analyze the effects of PA in mechanical inhibition of bone metastasis of 40 women with advanced breast cancer [91]. BC tumor microenvironment (TME) could also be affected by modifiable lifestyle factors, such as PA. TME is composed by a wide variety of different cell types, mechanical and chemical stressors, together with humoral factors. The interplay of these different components affects tumor cell characteristics and, subsequently, tumor growth rate and aggression [92,93]. Thus, PA may affect cell populations or its features. Gholamian et al. [94] described the role of PA on the decrease of mesenchymal biomarker gene expression, along with augmented muscle strength impeding cachexia. Exercise may also inhibit the recruitment of certain immune cells’ populations by the tumor, thereby favorably changing tumor microenvironment [63]. Combining PA with AOX supplementation prevented cachexia and muscle wasting and additionally decreased tumor volume in 4T1 breast cancer mice, through interacting with the immune system [95]. On the other hand, Jones et al. [96] demonstrated in vivo the impact of PA in the increased vascularization of BC tumors. Modulation of tumor hypoxia may be a possible explanation for these facts, as the relationship between exercise and a reduction in tumor hypoxia has been shown, hence reducing tumor growth [97]. In this line, Agostini et al. showed the powerful effect of PA in the inhibition of the hyperactivated PI3K pathway providing a potential target of BC [18,98]. In the same manner, PA is also responsible for a transient systemic acidosis, interrupting tumor adaptation to hypoxia and acidosis in the later steps of carcinogenesis [99]. These studies show the potential role of PA as a co-adjuvant therapy, particularly when combined with chemotherapy. Interestingly, these favorable effects of exercise appear to be beneficial, even at lower doses, and dose-independent, as higher levels of PA did not report differences in serum levels of growth factors when compared to lower exercise levels [100].

Overall, these mechanisms highlight the importance of PA in BC biology, as summarized in Figure 1, which for health professionals, may serve to promote exercise in BC patients.

## 4. Specific Considerations of Training Women with BC

When selecting exercises and routines, many factors must be considered, like the type of training, intensity, dose, or timing. On the other hand, it is critical to understand that rather than a unique or better routine, individuals’ sensations, motivation, and adherence to the training are the most important factors to consider when selecting the training program [101]. Main barriers to practice PA include physical (cancer-related symptoms), environmental (organization of time, lack of facilities or knowledge), and psychosocial (lack of motivation, low social support or self-confidence) [102]. Main motivators reported were body image, health improvements, and social support. It is critical for health professionals to communicate clear and detailed information of PA as soon as diagnosis is established [103]. Also, the previously mentioned barriers should be addressed, and further research is required to describe more suitable strategies for the BC population. In this context, psychosocial approaches may help in the management of PA motivation and adherence in BC patients and survivors [104,105,106]. Thus, each case should be analyzed individually to properly find the motivation of the women to practice PA. Hartman et al. [107] indicated that women with BC showed great attitudes regarding receiving personalized information and specific ways to individualize their trainings, as well as stories from other patients to achieve their motivation and adherence to the program.

Therefore, to assure the completion and maintenance of PA during and after BC treatment, it would be equally important to consider multiple determinants such as previous PA/sport practiced by the patient, the environment, her preferences, and interests. For instance, the Advanced Breast Cancer and Lifestyle Exercise Study (ABLE) trial showed that women with metastatic BC reported higher interest in training in a community sport center rather than from home [108]. Group vs. individual training would also be an interesting factor for both BC patients and survival, as some women have reported their preferences to training in a group rather than individually [109]. A recent systematic review [110] found that group interventions may provide more benefits when compared with individual exercise, but this conclusion cannot be confirmed due to the heterogeneity of the studies. Further research should be established in this field. Perhaps a combined individual and additional group training could maximize the benefits of PA. Multiple types of social PA like Pilates, dancing, or football have reported potential benefits in BC management during and after therapy [111,112,113], thus these programs could be equally helpful as additional sport training in women with BC. 

Keeping this in mind, different programs of exercises have shown their efficacy in BC management, with special focus on cardiorespiratory health, strength, and flexibility [114,115]. Aerobic or endurance training (AT) and strength or resistance training (RT) are the most important methods in BC management. AT is typically performed on a relatively slow load and during a long time, whereas RT includes higher loads and shorter durations. Although classically AT was associated with physical endurance and RT with muscle strength, it is now widely recognized that both trainings may overlap in their adaptations, with multiple benefits on the individual’s health [116]. In fact, evidence shows us that the combination of both AT and RT rather than individually is of greater interest in BC patients during and after therapy [117]. Two potential reasons are the role of both trainings combined in the cardiovascular health of the BC patient [118] or in reducing systemic inflammation [119]. In this line, Hiraoui et al. [120] reported substantial benefits of both AT and RT in women with BC in their cardiorespiratory responses along with perceived fatigue. Combination of AT and RT have also shown important benefits in the muscular strength and quality of life parameters, including upper-joint flexibility and mobility, fatigue, depression, life satisfaction, and other complications derived from BC [121]. Also, the potential benefits of these trainings could be even more important in patients with overweight, obesity, or other comorbidities, and adapted trainings could be needed for these women [119].

Dose and response to exercise represent a key factor in BC patients, as it would be vital to reach the maximum benefits, avoiding overexertion, which could be hazardous for the patient, also reducing motivation, adherence, and success of the exercise program. Different factors must be considered here, such as patient’s fitness or personal sensations. Nonetheless, the evidence shows that an excessive dose is not necessary to obtain the greatest effects. Consistent with these results, another clinical trial (NCT01435005) reported that higher volume of aerobic training (300 min/week) did not bring better results than public health recommendations (150 min/week), even for those women preferring greater volume of exercises [122]. Higher dose of training (50–60 min) of AT/AT-RT combined training does not report superior benefits than lower doses (25–30 min) of AT in a general BC population. Only patients with clinical depression at baseline showed more gains of higher dose of AT/AT-RT combined training [123]. It seems that dose of training does not represent an impacting variable in general BC patients when analyzing the benefits reported, remarking that the important point is practicing PA. However, for certain groups of women, especially with emotional needs, higher doses of trainings should be recommended.

Frequency, number of series, repetitions, and exercises are factors of training to consider when training BC patients. Some clinical trials have emerged to answer some of these important questions. The BEAUTY program (Breast Cancer Patients Engaging in Activity and Undergoing Treatment program) proposes a combined training of aerobic (2 days/week), resistance (1 day/week), and flexibility (5–7 days/week). Women can train from home or from a training center. They divide the work into hardest to easiest levels, depending on the patient’s sensations and progression, playing with the duration, sets, repetitions, and number of exercises [124]. Interestingly, they observed higher benefits from training 24 weeks in comparison to 12 minimum weeks in women with BC [125], hence denoting that wider duration of a PA program could maximize the success of PA intervention in women with BC. Optitrain (Optimal Training Women with Breast Cancer) (NCT02522260) aims to find the most adequate exercise regimens in patients with BC. In a 5-year follow-up cohort of 240 women with BC, different programs of training are compared, including a program evaluating the methodological approaches of training, AT alone (Group 1), both AT plus RT (Group 2), and patients receiving usual care (Group 3). Likewise, the effects of the programs will also be assessed in multiple parameters, including biological, clinical, and psychosocial outcomes, along with adherence and attendance rates to the different programs [126]. Order of exercises might also be important to maximize the impact of training, and some trials are starting to also consider this factor (NCT01157767).

Intensity is another component to consider in the programming of BC exercises. It is noteworthy that previous evidence suggests that high-intensity exercises would be considered in patients with established chronic diseases [127], as it could be for BC women. However, it is also important to adapt exercise training intensities in BC patients, as some guides for normal recommendations for healthy subjects cannot be applied in this population [128]. Determining patients’ perceptions and responses could be critical to find out whether high-intensity exercises would be beneficial or harmful to each woman. A systematic review conducted by López et al. [129] found that low-volume RT would be more suitable in BC patients receiving primary therapy, allowing gradual progression during the intervention. Thus, evidence shows us that a High-Intensity supervised Heavy Load program (85–90% one-repetition maximum (1RM), 3 sets of 5–8 repetitions) could improve some variables in comparison to low intensity, including muscle strength or reduction of chemotherapy-derived symptoms [130]. Interestingly, this study was carried out in physically inactive women, thus showing us that if motivated and guided, patients with BC are able to complete high-intensity trainings, with important improvements in their health. High-intensity interval training (HIIT) represents one of the most interesting types of intense PA, currently being the object of several clinical trials (NCT03679559; NCT02923401; NCT02883699). Mijwel et al. [131] demonstrated the effectiveness of high-intensity interval training (HIIT) in the management of patients with breast cancer. They compared the benefits of combined HIIT with resistance training (RT-HIIT), and with moderate-intensity aerobic exercise (AT-HIIT) with usual care, for 16 weeks. They observe that both AT-HIIT and RT-HIIT promote cardiorespiratory fitness and muscle strength, prevent weight gain and pain sensitivity, and accelerates return to work, although RT-HIIT could be a more interesting combination in these patients [132,133]. Equally, these programs were associated with lower rates of hospitalization and lower incidence of chemotherapy-related complications [134].

On the other hand, these results change in BC survivors. In the BC-PAL pilot study (NCT03564899), 45 survival women with BC practicing low- vs. high-intensity trainings and without intervention were compared. The results show that both low- and high-intensity present benefits looking at cardiopulmonary fitness, but only the low-intensity group presented reductions in sedentary time [135]. In this line, low- and moderate-, but not high-intensity training, were inversely associated with depression symptoms in BC survivors [136]. A possible explanation might be that, without personalized trainings, the adherence to high-intensity routines is harder to maintain. Hence, it would be interesting for BC patients after therapy to keep on training as previously, joining a gym or any activity that may motivate them to prolongate moderate–intense PA, which may have positive repercussions in their future.

**Figure 2:**
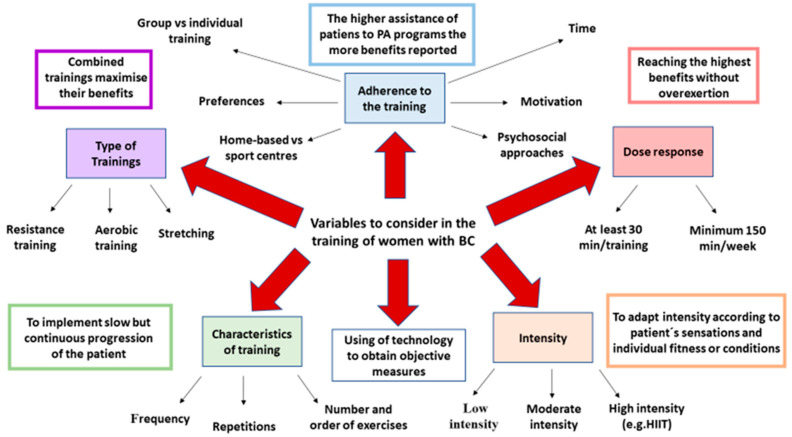
The multiple factors to consider in the training of women with BC. As described above, types of trainings, adherence to the program, dose-response effects, intensity, and variables of the training are crucial to understand and maximize the benefits of PA interventions. Using of technology may also aid in obtaining objective measures that together with subjective measures, motivation, and constancy, determine the basis of a successful training program.

It is important for trainers, patients, and researchers to implement current technologies, including activity trackers, accelerometers, or wearables to obtain objective measurements of PA complementary to subjective and personal perceptions [137,138]. Monitoring all the possible variables may help in the establishment of more effective exercise programs. To summarize, there are many factors to consider when training a woman with BC (Figure 2). Coordinated action of health professionals may be key to maximize the results of PA in these patients.

## 5. The Association of PA with BC Mortality and Survival

PA has been related with better prognosis and survival in BC. Johnsson et al. [139] studied 847 women aged 34 to 87 years old with BC, reporting a reduced all-cause mortality in the most active group. Importantly, their results showed that the improved survival of BC was only observed in women > 55 years, denoting the higher impact of PA in postmenopausal BC patients. A systematic review conducted by McTiernan et al. [140] found an inverse association between PA and all-cause mortality (up to 48% of risk reduction) and cancer-specific mortality (38%). PA may have a different impact in active women pre-diagnosis and post-diagnosis, with the last group being more sensitive to exercise. As shown by Jung et al. [141], a reduced risk of overall mortality (OM = 0.50), BC-related mortality (BCM = 0.54), and recurrence-free survival (RFS = 0.58) was observed in increasingly active women, whereas for decreasingly active women and sufficiently active women, less benefits were reported (OM = 0.91, BCM = 0.80, RFS = 1.04; OM = 0.75, BCM = 0.61, RFS = 0.80, respectively). Also, Palesh et al. [142] showed that patients with advanced stage IV cancer practicing moderate PA ≥ 1 h per day presented a 24% reduced mortality and longer survival than those with reduced PA (<1 h). A recent meta-analysis conducted by Lee [143] found an inverse association between PA with all-cause and breast cancer-specific mortality in women getting ≥300 min per week of moderate PA. Nonetheless, decreased BC mortality is observed from 3 h walking, and there is no evidence that vigorous exercise improves the survival [144]. On the other hand, the DELCaP (Diet, Exercise, Lifestyle, and Cancer Prognosis) study found, in 1340 women with BC, that meeting the minimum guidelines before and after treatment was directly associated with increased survival and favorable prognosis, and a different hazard of mortality according to the dose of PA (Low = 0.41, Moderate = 0.42, and High = 0.31) [145]. Importantly, women who exceed these recommendations did not obtain further benefits when compared to low volume of regular activity in terms of overall survival. Before treatment, muscle strength, but neither muscle mass nor radiodensity, was associated with better prognosis and longer survival [146]. The presence of comorbidities could also affect BC prognosis, along with increased mortality [147]. PA may aid in the gain of muscle strength and management of BC comorbidities, but it has been demonstrated that patients with both reduced muscle strength and with comorbidities are related with physical inactivity, contributing to the worsening of prognosis and survival [148]. Likewise, PA can contribute to weight management, which is, in turn, associated with better prognosis in women with BC [149]. Overall, these studies elucidate the importance of implementing PA programs in all BC patients, as it could positively impact on the survival, reduced mortality, and prognosis of BC patients. 

## 6. PA in BC Management

Although BC therapies have provided substantial improvements in BC treatment, they might be associated with a broad spectrum of complications, which instead will affect the QOL of BC patients. Among them, chemotherapy is the most strongly associated treatment with the decrease on QOL of BC women [150], so this group of patients will be positively influenced by interventional changes. Despite the fact that pharmacological interventions are frequently used to ameliorate temporarily certain symptoms, PA and behavioral management seem to be the most effective approaches to possibly address the underlying etiology of treatment-derived complications [151,152]. Here, we will summarize the evidence of PA prescription in the management of BC-related adverse effects, including cardiovascular complications, lymphedema, body composition, peripheral neuropathy, and cognitive dysfunction.

### 6.1. PA and Cardiovascular Disease in BC

BC and cardiovascular disease (CVD) are conditions directly correlated. In fact, it is estimated that 1.6% to 10.4% of BC mortality is due to CVD [153]. Firstly, both conditions show different overlapped risk factors, including non-modifiable (age, race, family history) or modifiable factors (smoking, diet, physical inactivity, or overweight). Last but not least, BC treatments may also be responsible for CVD in these women [154]. Radiotherapy, and prominently, many chemotherapeutic agents, are considered potential risk factors of CVD in BC [155]. Anthracyclines and Herceptin are common treatments in BC, but they are also responsible for CVD among these women. Approximately 29.6% of patients with BC received anthracycline therapy, 0.9% received trastuzumab alone, and 3.5% received anthracycline plus trastuzumab [156]. Herceptin-related cardiotoxicity is not dose-dependent (Type II cardiotoxicity), being often reversible after treatment disruption. Conversely, Anthracyclines cardiotoxicity is dose-accumulative, non-reversible cardiotoxicity (Type I) [157]. It has been demonstrated that PA prevents the deleterious effects of these agents during the treatment, mainly by its cardioprotective effects through the improved systemic antioxidant capability of PA in the heart and blood vessels to compensate the increased oxidative stress derived from the chemotherapy [158,159]. Different clinical trials, such as TITAN (NCT01621659) (Multidisciplinary Team IntervenTion in cArdio-oNcology), are being conducted to assess the influence of multidisciplinary healthcare, including PA, on the prevention of cardiovascular harm and other adverse outcomes versus conventional medical care [160]. Furthermore, another clinical trial (ISRCTN32617901) is currently taking place to demonstrate the impact of PA (3 days per week with progressive volume and intensity during the period of treatment) on anthracycline-derived cardiotoxicity medical care. Multiple variables will be studied, including biochemical, physiological, and psychological parameters [161]. Even from an acute dose, PA seems to prevent and improve cardiovascular function prior to chemotherapy, as demonstrated by Kirkham et al. [162], who observed a wide multitude of positive effects of 30 min duration PA only 24 h before doxorubicin treatment, including echocardiographic, serological, and physiological parameters.

In the case of Trastuzumab, previous research shows that this antibody promotes cardiac remodeling, independently of practicing AT [163]. However, low adherence to the program of training may explain these results. More interestingly, fitness capacity, or VO_2max_, is a predictor of anthracycline- and trastuzumab-induced left ventricular dysfunction and CVD risk in BC patients [164]. The CARDAPAC (Physical Activity Intervention on Myocardial Function in Patients with HER2 + Breast Cancer) study (NCT02433067) aimed to study the effect of PA in patients treated with this therapy, consisting of supervised intermittent exercise with the combination of moderate and high intensities: 55 min duration, 3 times per week [165].

Endocrine therapy (ET) is a common treatment for ER-positive BC. However, both derivates, tamoxifen and AI, are related with the incidence of CVD [166]. PA is a potential opportunity in the management of ET-related side effects, particularly in young women [167,168]. In patients older than 65 years, PA increased overall survival in patients receiving ET [169].

Radiotherapy is associated with an important cardiopulmonary loss, probably due to its effects in chest wall restriction [170]. Nevertheless, PA is well-tolerated and even shows beneficial results after radiation therapy, counteracting different radiation-related side effects [171,172]. For instance, PA decreases the levels of the kynurenine pathway, with carcinogenic effects [173]. A systematic review and meta-analysis conducted by Shen and Yang [174] demonstrated remarkable improvements of PA on the physical capacities and other parameters in women after radiation therapy. However, further studies should be directed to unravel adequate exercises to maximize their benefits in cardiovascular health [175].

### 6.2. PA and Lymphedema

BC-related lymphedema (BCRL) affects around 1 in 5 women with BC during or after treatment [176]. Axillary surgery, lack of breast reconstruction, or some adjuvants like taxane-based chemotherapy are potential risk factors of BCRL [177,178]. High Body Mass Index, subclinical oedema, and cellulitis are considered non-treatment-related risk factors [176]. Low PA and age are the main causes of BCRL functional problems [179]. Thus, PA clearly represents a potential solution for patients at high risk of BCRL. A systematic review conducted by Baumann et al. [180] that included 458 BC patients showed, independently of the type of exercise practiced, both objective and subjective improvements in women with BCRL. For instance, the association among diverse modalities of PA has been described, including yoga, walking, or RT, which were shown to reduce oedema volume in BCRL [181].

Interventional weight loss is considered an effective approach to reduce BCRL [182]. To answer the relationship between PA, weight, and lymphedema, a home-adapted training combined with weight loss multidisciplinary management clinical trial WISER (Women In Steady Exercise Research) was conducted in 351 overweight patients [183]. However, they did not obtain significative improvements in women with BCRL after completing their training programs, hence concluding that a supervised facilities-based program may result more interesting in these patients [184]. Similarly, home-based exercises in combination with aqua lymphatic therapy led to a reduction in pain and arm disability caused by BCRL, while home-based exercise alone did not report any variation in BCRL symptoms [185]. In patients at risk or with BCRL, it would be important to carry out personalized, effective treatments to maximize the benefits of PA in this group. Sustained PA before and after intervention, along with simple lymphatic drainage, may be used to prevent BCRL after surgery [186]. On the other hand, lack of knowledge about safety and effectiveness of practicing PA is a major concern in patients with BCRL [187]. No studies report any adverse effects of PA in BCRL, even at higher intensities and heavy weights [188,189]. This is a crucial point, as evidence demonstrates that PA, apart from its multiple benefits, is completely safe for women presenting BCRL. Clinicians should inform patients at risk or with BCRL the necessity and the safety of performing PA, as it may encourage patients to practice PA.

### 6.3. PA and Body Composition in BC

Women presenting with BC also have notable impairments in body composition due to the therapies received. Chemotherapy provokes marked changes in body composition of BC patients, including a reduction in the muscle mass and strength, with increased body fat percentage, particularly in premenopausal women [190,191]. A recent meta-analysis showed that chemotherapy is associated with weight gain, remarkably in women under cyclophosphamide, methotrexate, and 5-fluorouracil (CMF) regimes [192]. Multiple studies have denoted that physical inactivity during chemotherapy is a major contributor to weight gain in BC patients [193,194].

Muscle of women with BC presents multiple metabolic disruptions at various levels, comprising oxidative phosphorylation, mitochondrial dysfunction, peroxisome proliferator-activated receptor activation, and IL-15 signaling, a cytokine which appears to be associated with chronic fatigue, along with IL-6, also augmented by chemotherapy [195,196]. These changes in body composition seem to also be promoted by decreased levels of IGF-1 [196]. Importantly, it seems that muscle mass loss occurs throughout the treatment, although the impact of the therapy will depend on the number of cycles completed [197]. PA could be an ideal tool in the management of all these disturbances. A systematic review conducted by Stene et al. [198] demonstrated that both AT and RT alone or combined may report significant improvements in muscle strength, although RT seems to be more adequate. Although RT presents multiple benefits and it is more preferred by women during BC therapy, it may have harmful effects on satellite cells, aggravating the condition. RT stimulated entrance in the cell cycle and proliferative mechanisms to facilitate muscle repair, regeneration, and hypertrophy. Nonetheless, chemotherapy frequently attacks rapidly dividing cells, which may be implicated in accelerating muscle aging of the patient [199,200]. Recently, a novel clinical trial has been carried out to assess the effect of heavy-load RT during chemotherapy on muscle parameters, including (NCT03945838) the status of these satellite cells. Further studies are needed to evaluate the impact of long-term RT. 

However, the little data that are available regarding muscle strength in advanced BC patients or at risk of cachexia, for instance, have proven the maximum effects in improving muscle strength while reducing inflammation and fatigue [201]. RT-HIIT was shown to be an effective combination program to increase muscle strength and decrease fatigue in a cohort group of 206 women with BC, in comparison to those receiving usual care [202]. Multimodal exercise interventions including AT, RT, and stretching training are also associated with improvements in cancer-related fatigue [203]. Although effective in increasing muscle strength, less evidence supports the use of RT when looking at body composition [204]. To describe in detail the potential effects of PA in BC changes, the COBRA study (Change Of Body composition in Breast cancer: All-in Assessment-study) used a mixed methods designs to obtain both quantitative measures of changes in body weight, body composition, as well as lifestyle factors and qualitative assessments of women´s perceptions, compared with non-cancer women [205]. Adapted exercises and physiotherapy intervention could also be associated with enhanced shoulder ROM (Range of Movement), a common BC complication after surgery [206,207].

On the other hand, the importance of exercise in the bone mass density has been demonstrated. Certain agents such as AI are also correlated with bone loss and osteoporosis risk in BC patients [208]. All patients starting with AI therapy should be recommended to exercise moderately, and RT has proven the maximum benefits in these patients (Evidence level 1A) [209]. In fact, patients with more adverse effects during AI therapy reported a reduction in their levels of PA [210]. Winters-Stone et al. [211] described a program based on RT exercises, showing an augmented muscle strength while avoiding bone loss, with greatest effects in BC survivors who received AI. Importantly, Tabatabai et al. [212] found that the beneficial effects of RT in bone density only occurs in patients who did not report muscle mass loss, but keeping muscle mass by itself did not prevent bone alterations. This means that combined RT plus an adequate muscle mass are the best approaches to maintain bone density in patients with BC.

### 6.4. PA and Peripheral Neuropathy

Chemotherapy-induced peripheral neuropathy (PN) refers to a progressive, persistent, and sometimes irreversible condition that affects approximately 30–40% of patients with BC receiving chemotherapy, including paclitaxel, docetaxel, cisplatin, carboplatin, bortezomib, or vincristine [213,214]. This condition provokes structural deficits in Dorsal Root Ganglia, affecting sensory neurons in this region, satellite cells, Schwann cells, as well as neuronal and glial cells in the spinal cord. Many biological mechanisms occur, including excitotoxicity, oxidative stress, or mitochondrial dysfunction [215]. PN causes important symptoms, such as sensory loss, paresthesia, or dysesthesia, with negative consequences for the patient and success of therapy. Sensory neurons in the Dorsal Root Ganglia are the preferential sites in which chemotherapy neurotoxicity occurs. Although very rarely, radiotherapy may also induce PN in BC patients [216]. PA may positively influence clinical management of PN in patients receiving taxane, platinum, or vinca alkaloid-based chemotherapy [217]. It is well-known that PA is an important tool against PN, due to its negative modulatory effects on inflammation, pain, sensory, and motor dysfunction [218]. Twelve weeks of supervised training attenuated symptoms of PN, improving overall QOL [219]. Ten weeks of home-based exercises also reported the same benefits in reducing PN-related symptoms [220]. Not only AT and RT may be useful in BC patients presenting PN. Sensorimotor training has been described as an interesting training in order to improve balance performance, altered by PN [221]. Programs including core and pelvic girdle strength are recommended approaches to train balance in this population [222]. However, more doubts remain when studying the effect of RT. It seems that moderate RT, along with sensorimotor exercise, may aid in the prevention of muscle loss and clinical outcomes [223]. On the other hand, other PA activities such as yoga may result beneficial for patients with PN associated to BC treatment (NCT04075097). 

### 6.5. BC-Related Cognitive Dysfunction and PA

Cognitive dysfunction is a common adverse outcome in BC patients, with important alterations in the attention, processing speed, memory, and executive functions [224]. BC-related cognitive dysfunction is experimented in nearly 50% of patients, being worryingly related with psychological disorders, such as depression, anxiety, stress, or mental fatigue, among others, therefore importantly limiting QOL of BC [225,226]. Chemotherapy and its neurotoxic effects are partly responsible for the cognitive impairment after treatment, what is known as “chemobrain”, but also, other non-chemotherapy-related factors, such as age or even genetic polymorphisms, may play a prominent role in the cognitive dysfunction defined in BC patients [227,228,229]. PA is a key modulator of brain function and directly influences on cognitive processes and memory, presenting analgesic and antidepressant effects, acting as potential inductors of well-being after the production of certain substances as endorphins [230]. As part of the cognitive decline, different hallmarks of aging have been found in the brain of patients with BC, including attrition of telomeres, mitochondrial dysfunction, genome instability, epigenetic alterations, cellular senescence, and altered intracellular communication, along with exacerbated inflammatory response [231,232]. PA has a great potential as a non-pharmacological intervention to regulate BC-related cognitive dysfunction. Of note, not all the domains of cognitive function may be equally affected by PA, as some of them, such as executive function, could be more likely to positively change with PA [233].

However, a meta-analysis conducted by Furmaniak et al. [234] in BC patients receiving adjuvant therapy showed that although PA may have positive effects in BC-related cognitive dysfunction, there is still a long road to find the optimal type, intensity, and timing of an exercise. An intervention carried out by Campbell et al. [235] of 24-week AT found improvements in the processing speed test and functional changes in several brain regions of interest. This occurs even at acute doses of AT, where memory and processing speed was improved in BC women [236]. However, they did not find differences between self-reported cognitive functions and attention, and the sample size of the studies was not high enough. Mindfulness-based PA, such as yoga and Qigong, may aid additional exercises in their potential benefits looking at cognitive functions, although further research is needed to find the proper dose, content, and timing of these interventions [237,238,239].

Northey et al. [240] compared the effect of moderate-intensity continuous training with HIIT in BC survivors. Interestingly, they found that despite the fact that both were associated with improvements in cognitive functions, HIIT showed larger benefits in some cognitive properties, such as executive function, episodic, or working memory, along with positive cerebrovascular outcomes including cerebral blood flow and cerebrovascular reactivity. Nonetheless, the sample was not high enough (*n* = 17), so further studies are needed to demonstrate the benefits of HIIT in brain health. With that purpose, a clinical trial named the Chemobrain in Motion [241] study aims to investigate the potential effects and benefits of HIIT on breast cancer-related cognitive dysfunction. So, further research will shed light on the effects of HIIT and PA interventions in cognitive function on BC. Moreover, most studies are conducted in BC survivors, but little information is available regarding BC patients during and before treatment [233]. However, current research support that both combined optimal diet and PA report enormous effects in patients with BC undergoing therapy [242], and it should be implemented as soon as possible. Therefore, further research is required in these fields, as it would bring important benefits in the cognitive functioning, health-related outcomes, and QOL parameters. Some of the most relevant PA concepts regarding cognitive dysfunction and therapy-related adverse effects are summarized in Table 1.

## 7. Physical Activity in the QOL of BC Patients

Diagnosis and treatment of BC are also related with a prominent decrease in the QOL of these women. Firstly, it is difficult to establish a proper definition of QOL. The description of health exposed by the World Health Organization (WHO) in 1948 as the “state of complete physical, mental and social well-being, and not merely the absence of disease and infirmity” [243], has brought significant confusion about the concept of QOL and the previously stated term “well-being”, and little consistent data is found in the literature regarding this term [244]. Nevertheless, Spitzer’s QL index was the first QOL measure, based on socio-personal variables including physical, social, and emotional function, personal features of patient’s daily lives (involving familiar and interpersonal interactions), attitudes, and the cost of illness [245]. Questionnaires such as EORTC QLQ-BR23 (European Organization for Research and Treatment of Cancer (EORTC) Breast Cancer-Specific Quality of Life Questionnaire BR23) and the FACT-B (Functional Assessment of Cancer Therapy-Breast Cancer) are some of the best methods to measure QOL in BC patients [246]. Based on these surveys, near 60% of women reported a prolonged decrease in their QOL even after treatment completion [247]. Self-confidence, disease duration, other comorbidities, lifestyle, social support, marital satisfaction, caregiver status, and unmet needs are some of the most important predictors of the QOL of BC patients [248]. Emotional and social functioning, along with functional role, are the most affected domains in BC patients, with pain, fatigue, and insomnia as the main influences of these disruptions [249]. Increased risk of depression, anxiety, suicide, and neurocognitive or sexual dysfunctions have also been reported in BC survivors when compared to non-cancer women [250]. To increase QOL of BC patients and survivors, many lines of research are opened, prominently from a multidisciplinary approach. PA could represent a potential ally to help patients with BC to present substantial improvements in their QOL, which will influence the clinical management of BC patients and survivorship [251]. Women receiving adjuvant chemotherapy participating in a 16-week duration PA intervention remarked the great impact on their physical function and mental wellbeing, along with increased feeling of social support, positively influencing the adherence and motivation to PA [252]. Some trials have arisen to better study the effect of PA interventions in the QOL of BC patients. Breast cancer and Exercise (BREX) investigated the diverse impacts of a 5-year cohort training initiated shortly after treatment to the free-survival period to study the QOL of these women. Importantly, their results highlighted a prominent, sustained improvement in their global health score, social and role functioning, body image, and even perspectives of the future [253]. The AMBER (Alberta Moving Beyond Breast Cancer) study enrolled 1500 women with I-IIIc BC and followed them in a 5-year cohort, to measure the influence of PA and health-related fitness objectively and subjectively in long-term QOL, treatment completion, alleviation of treatment adverse effects such as lymphedema, or even in the secondary prevention of BC [254]. Their preliminary sample of 500 patients showed a positive attitude and willingness in women with BC to practice PA from the moment of diagnosis, especially in those with overweight or obesity (60% of total), which may provide substantial changes in their QOL [255]. Physical exercise is equally associated with improved physical functioning and reduction of fatigue after a 6-month follow-up, as demonstrated in a meta-analysis collected by Juvet et al. [256].

Sleep disturbances are frequently associated with BC at pre-diagnosis (25%) and postdiagnosis (approximately 50%), with some of these patients presenting insomnia [257]. PA and sleep have a bidirectional relationship in which both may influence each other [258]. However, these interactions are multifactorial, and it seems that sleep is better associated with next-day performance PA, rather than PA is associated with sleep [259]. In this line, Bernard et al. [260] showed that management of insomnia could increase PA practice in BC patients, although a reciprocal relationship cannot be discarded. Different strategies could be followed in the management of insomnia, including psychosocial interventions, behavioral therapy such as CBT-I (Cognitive behavioral therapy for insomnia), or yoga and mindfulness programs [261]. Melatonin could also be of aid as it brings subjective improvements in the quality of sleep, with no adverse effects [262]. Therapies received such as ET, discomfort from local therapy, and pain and fear of recurrence could promote BC-related insomnia [261]. On the other hand, PA may have important impacts in BC survivors. Rogers et al. [263] showed that PA was associated with self-reported improvements in quality of sleep, not objectively detected, resulting from the multiple benefits of PA in BC. 

Emotional aspects like depression or anxiety may also be favored by PA. Physical changes caused by some procedures such as radical mastectomy or cytotoxic therapy, posttraumatic stress, or fear of cancer recurrence, may be related with the appearance of depression and anxiety [264,265,266]. A meta-analysis conducted by Zhu et al. [267], with 33 controlled trials included and a total sample of 2659 BC survivors, found a substantial improvement in women practicing PA regarding their QOL, particularly in their mental and general health, along with emotional and social well-being with reduced depression and anxiety. Despite its multiple benefits, depression symptoms may result in an altered self-perception of the difficulty of exercise, which may affect the likelihood of a BC patient to engage a PA [268]. Thus, different strategies should be established to help these women to maintain PA levels. Recently, Ribeiro et al. [269] identified the importance of leisure time/community in the management of both symptoms rather than occupational or participation in a sport/exercise. If practiced in company or in their free time, women with these symptoms could be more susceptible to initiate and maintain PA. Psychological interventions could be equally recommended to maximize and assure PA benefits in these groups of patients [101].

## 8. Conclusions and Future Directions

As extensively reviewed, PA brings a wide multitude of positive effects in BC. PA plays a pivotal role in the prevention of this condition, along with a proper diet and weight control. Through epigenetic mechanisms, PA controls numerous biological mechanisms, including sexual and metabolic hormones, immune system, or oxidative stress. Likewise, PA also interacts directly with the tumor and its microenvironment, which may promote its use as an additional co-adjuvant therapy. What is more, PA might also provide an important support during BC therapy, improving the different complications derived from the treatment. This could lead to a substantial gain in the QOL of BC patients, as well as its relationship with other parameters, including fatigue, pain, insomnia, and social and emotional functioning. Because of that, PA prominently affects BC prognosis and survival. However, there are still some challenges to address regarding the training of women with BC. From the beginning, health professionals should become aware of the important role of PA in these patients in order to achieve the previous reported benefits. Many research and clinical trials are established to find the adequate program for BC. Evidence shows us that combined AT and RT are great options for these women, together with stretching, balance, and mindfulness PA. Different intensities, doses, or frequencies of training may be important variables that should be taken into account. Each patient must be analyzed individually, not only looking at her physical fitness, but also her motivation, time, possible complications, subjective and objective QOL, training from home or not, individually or collectively, etc. The use of current available technology may be of aid to better control the different variables. Sustained adherence and motivation is the most important objective of any PA program, as exercise is always beneficial for patients with BC, BC survivors, and to prevent this condition in healthy women. It is essential to understand that PA is another part of a great and complex puzzle. PA, along with other lifestyle interventions, did not cure BC by itself, but it entails an imperative additional support to maximize the rate of success in BC management.

## Figures and Tables

**Table 1:** Potential examples and mechanisms of Physical Activity (PA) benefits in different therapies-derived adverse effects.

Complications	Etiological Care Agent	Favorable Actions Mediated by PA	Interventions	Main Objectives	References
**Cardiovascular diseases (CVD)** Approximately 2–10% of BC mortality	Shared risk factors including age, sex, smoking, diet…	Substantial biological benefits from PA (Hormone-levels, antiaging, organokines, etc.)	TITAN study	Multidisciplinary approaches to reduce risk of CVD including from cardiology, clinical nutrition, psychology, PA and physical therapy	NCT01621659
	Chemotherapeutic agents mainly trastuzumab, anthracyclines or ET	Cardioprotective effects (Counteracting the oxidative stress derived from chemotherapy in the cardiovascular system)	Effects of physical exercise on cardiac health in women with breast cancer	To assess the impact of 3 days per week with progressive volume and intensity during the period of anthracycline treatment	ISRCTN32617901
	Radiotherapy	Promotion of cardiovascular health	EMBRACE study	To evaluate the potential role of PA in the prevention of chemotherapy-derived cardiotoxicity.	NCT04467411
**Breast** **Cancer-Related Lymphedema (BCRL)** 1 in 5 women will develop this condition	Axillary surgery, lack of breast reconstruction or some adjuvants like taxane-based chemotherapy	PA reported both objective and subjective improvements in patients with BCRL	WISER study (Women In Steady Exercise Research)	To observe the impact of one-year weight loss and exercise in sedentary overweight or obese breast cancer survivors with breast cancer-related lymphedema. Finally, they concluded that home-based training was ineffective for these women	NCT01515124
	Non-treatment-related factors (BMI, cellulitis, subclinical oedema	Even RT is safe and completely recommended for patients with BCRL	PAL trial (Physical activity and lymphedema)	Demonstrated the safety and efficacy of RT in BC patients with lymphedema	NCT00194363
**Body composition** Including weight gain, increased adiposity, reduced muscle mass, strength, and flexibility, along with bone mass	Chemotherapy (depending on cycles received or certain types of agents such as cyclophosphamide, methotrexate, and 5-fluorouracil (CMF)	RT and AT combined or RT-HIIT improves muscle strength and mass-reducing adiposity, inflammation, and fatigue	COBRA study	To investigate changes in body composition (adipose and muscle tissue) of women with breast cancer treated with or undergoing chemotherapy.	OND1350462
	Surgery	PA to promote range of movement of shoulder	Effects of exercise training at different timelines on shoulder dysfunction after BC-modified Radical mastectomy	To describe the most effective routines to deal with shoulder dysfunction after surgery, including isometric shoulder training with RT	NCT03658265
	ET is strongly associated with bone mass loss	RT may collaborate to maintain bone density in women receiving ET	Breast cancer and Exercise (BREX)	Proved the efficacy of regular exercise in the prevention of bone loss and enhancement QOL	NCT00639210
**Peripheral** **neuropathy** Affecting 30–40% of patients during chemotherapy and, in some cases, after this	Chemotherapy Paclitaxel, docetaxel, cisplatin, carboplatin, bortezomib, or vincristine	Attenuation of peripheric neuropathic symptoms by negatively modulating inflammation, pain, sensory, and motor dysfunction	Pain, psychological, and endocannabinoid responses to yoga in breast cancer survivors with chemotherapy-induced neuropathic pain	Combined yoga plus AT as a strategy in BC survivors to deal with peripheral neuropathy	NCT04075097
**Cognitive** **dysfunction** Up to 50% of patients with BC may impair its attention, processing speed, memory, and executive functions	Chemotherapy “chemobrain” plus other factors (age, genetics, etc.)	PA directly influences cognitive processes and memory, presenting analgesic and antidepressant effects, acting as potential inductors of well-being	Physical activity and neuropsychological outcomes in a cancer population	To demonstrate if PA also results beneficial for BC patients suffering cognitive dysfunction	NCT02332876
			Chemobrain in Motion	To evaluate the adequacy of HIIT in the cognitive functioning of BC patients	DRKS00011390

## Abbreviations

PA	Physical activity
BC	Breast cancer
TNBC	Triple-negative breast cancer
RT	Resistance/strength training
AT	Aerobic/endurance training
HIIT	High-intensity interval training
QOL	Quality of life
CVD	Cardiovascular disease
BCRL	Breast cancer-related lymphedema
PN	Peripheral neuropathy
